# Higher order photoprotection mutants reveal the importance of ΔpH-dependent photosynthesis-control in preventing light induced damage to both photosystem II and photosystem I

**DOI:** 10.1038/s41598-020-62717-1

**Published:** 2020-04-21

**Authors:** Roberto Barbato, Luca Tadini, Romina Cannata, Carlotta Peracchio, Nicolaj Jeran, Alessandro Alboresi, Tomas Morosinotto, Azfar Ali Bajwa, Virpi Paakkarinen, Marjaana Suorsa, Eva-Mari Aro, Paolo Pesaresi

**Affiliations:** 1 Department of Sciences and Innovation Technology, University of Eastern Piedmont Amadeo Avogadro, I-15121 Alessandria, Italy; 2 Department of Biosciences, University of Milan, I-20133 Milan, Italy; 3 Department of Biology, University of Padua, 35121 Padova, Italy; 4 Molecular Plant Biology, Department of Biochemistry, University of Turku, SF-20520 Turku, Finland

**Keywords:** Plant sciences, Non-photochemical quenching, Photosystem I, Photosystem II

## Abstract

Although light is essential for photosynthesis, when in excess, it may damage the photosynthetic apparatus, leading to a phenomenon known as photoinhibition. Photoinhibition was thought as a light-induced damage to photosystem II; however, it is now clear that even photosystem I may become very vulnerable to light. One main characteristic of light induced damage to photosystem II (PSII) is the increased turnover of the reaction center protein, D1: when rate of degradation exceeds the rate of synthesis, loss of PSII activity is observed. With respect to photosystem I (PSI), an excess of electrons, instead of an excess of light, may be very dangerous. Plants possess a number of mechanisms able to prevent, or limit, such damages by safe thermal dissipation of light energy (non-photochemical quenching, NPQ), slowing-down of electron transfer through the intersystem transport chain (photosynthesis-control, PSC) in co-operation with the Proton Gradient Regulation (PGR) proteins, PGR5 and PGRL1, collectively called as short-term photoprotection mechanisms, and the redistribution of light between photosystems, called state transitions (responsible of fluorescence quenching at PSII, qT), is superimposed to these short term photoprotective mechanisms. In this manuscript we have generated a number of higher order mutants by crossing genotypes carrying defects in each of the short-term photoprotection mechanisms, with the final aim to obtain a direct comparison of their role and efficiency in photoprotection. We found that mutants carrying a defect in the ΔpH-dependent photosynthesis-control are characterized by photoinhibition of both photosystems, irrespectively of whether PSBS-dependent NPQ or state transitions defects were present or not in the same individual, demonstrating the primary role of PSC in photoprotection. Moreover, mutants with a limited capability to develop a strong PSBS-dependent NPQ, were characterized by a high turnover of the D1 protein and high values of Y(NO), which might reflect energy quenching processes occurring within the PSII reaction center.

## Introduction

Photoinhibition of photosynthesis is a long-known phenomenon^1^. Due to the discovery of the high turnover D1-protein^2^ and its subsequent recognition as a main component of PSII reaction center harboring most of PSII redox cofactors^3,4^, photoinhibition was thought as the increase of degradation rate for the D1 over its synthesis or, more in general, as an unbalance between damage and repair of PSII^5,6^. The repair cycle of PSII is now a well-established phenomenon in which damaged PSII centers, localized in the grana domain, undergo monomerization, partial dismantling and lateral migration to the stroma-exposed regions, where newly synthesized D1-proteins, co-translationally inserted in the thylakoid membrane, substitute the damaged ones; reassembly of repaired centers, back migration to grana membranes and dimerization close the cycle^6–8^. Post-translational modifications of some subunits, such as reversible phosphorylation of D1, D2, CP43 and PSBH subunits, may have a regulative role in the process^9^.

Although photoinhibition has since long been considered as a process dealing with PSII, it is now clear that, under some conditions, also PSI may be highly vulnerable to light. Among them, irradiation with high light in the cold (at least in chilling-sensitive species), was first described as a condition affecting PSI. Electrons from PSI could generate superoxide radicals which, once reduced to hydrogen peroxide, could react with iron-sulfur centers on the acceptor side of the PSI, producing irreversible damages^10^. In addition, mutations such as *pgr5*, in which the ability to develop a full ΔpH trans-thylakoidal gradient is impaired, make PSI very sensitive to light, both in growth chamber^11,12^ and field conditions^12^, because of over-reduction of electron acceptors^13^. Owing to the absence of high turnover rates for PSI subunits, damages to this photosystem is very dangerous for plants. In this kind of mutants, PSII was also found to be highly vulnerable to light^14^, to the extent that PSII damage/repair cycle has been proposed as a photoprotection mechanism for PSI^15^.

Plants possess a number of different mechanisms able to regulate the use of light^14^. Excess of absorbed light is thermally dissipated in a process defined as non-photochemical quenching (NPQ) which, in turn, comprises a number of different processes. Among them, the principal, fastest and more investigated is qE (energy-dependent quenching), whose formation depends on low luminal pH, synthesis of zeaxanthin through the xanthophyll cycle, protonation of PSBS protein and participation of Lhcb proteins^16,17^. Depending on the redox state of Q_A_, LHCII may be phosphorylated by the STN7 kinase and act as an antenna also for PSI, re-equilibrating the distribution of light between the two photosystems^18,19^, resulting in a quenching of fluorescence known as qT^20^. In addition, inactivated PSII centers could act as quenchers, the extent of which is defined as qI^21^.

The trans-thylakoidal pH gradient is formed by the action of the linear electron transport (LET) and the cyclic electron transport (CET). In LET, electrons released from water in PSII are transferred to NADP/Ferrodoxin *via* PSI, with cytochrome *b*_6_*/f* connecting the two photosystems and generating ΔpH used for ATP synthesis. In CET, electrons may be recycled from NADPH or ferredoxin to plastoquinone and then to cytochrome *b*_6_*/f*, producing ΔpH which can be used to synthesize ATP without accumulation of reduced species. As stated above, ΔpH is essential for the activation of NPQ; it is also essential for photosynthesis-control, *i.e*. the slowing down of electron transfer from cytochrome *b*_6_*/f* to PSI, which limits the amount of electrons reaching PSI. In mutants with defects in ΔpH formation, such as *pgr5* (not able to photo-accumulate P700^+^), not only PSI but also PSII is very sensitive to high light^22^. As a consequence, the PGR5 protein should be regarded also as a component of the photoprotective machinery. Whether CET itself has a role in photoprotection is still a matter of discussion. Cyclic electron transport is composed of two pathways, the first one depending on the PGR5/PGRL1 complex sensitive to Antimycin A, the second depending on NADH-deyhydrogenase, insensitive to Antimycin A. While mutants impaired in the PGR5/PGRL1 pathway cannot accumulate P700^+^ in the light, NDH mutants such as *crr2-2, crr-3, crr4-2*^23^ and *crr4-3*^24^ can, with the former being more light sensitive than the latter^25^. Moreover, it should also be reminded that PGR5/PGRL1 proteins could affect the ΔpH regulation also in a CET-independent pathways^26^.

Although photoprotection is considered as a mechanism preventing PSII from photoinhibition, its relationship with D1 protein turnover is far from clear. Even less clear is the relative importance of the different described mechanisms in protecting PSII from photoinhibition, also through the high-turnover of the D1 protein.

In this paper, we analyzed light sensitivity of mutants carrying defects on the photoprotective PSBS-dependent NPQ (*npq4-1*), defects on trans-thylakoidal pH gradient formation (*pgr5, pgrl1ab*), and defects on the regulatory mechanism of state transitions (*stn7 stn8*) which was then compared with that of higher order mutants, in which PSBS-dependent NPQ, ΔpH formation and state transitions were depleted in different combinations. We found that light sensibility was higher when the ability to form ΔpH was impaired, irrespectively of whether PSBS-dependent NPQ or STN7-dependent state transitions were operative or not, indicating that the ΔpH, possibly by means of photosynthesis-control mechanism, is one main short-term photoprotective mechanism in leaves of higher plants. In addition, photoinhibition of PSII appears to be a further strategy to limit PSI damage and balance the activity of both photosystems.

## Results

### Arabidopsis plants devoid of short-term regulatory mechanisms highlight the primary importance of ΔpH-dependent photosynthesis-control for optimal PSI activity

**Figure 1:**
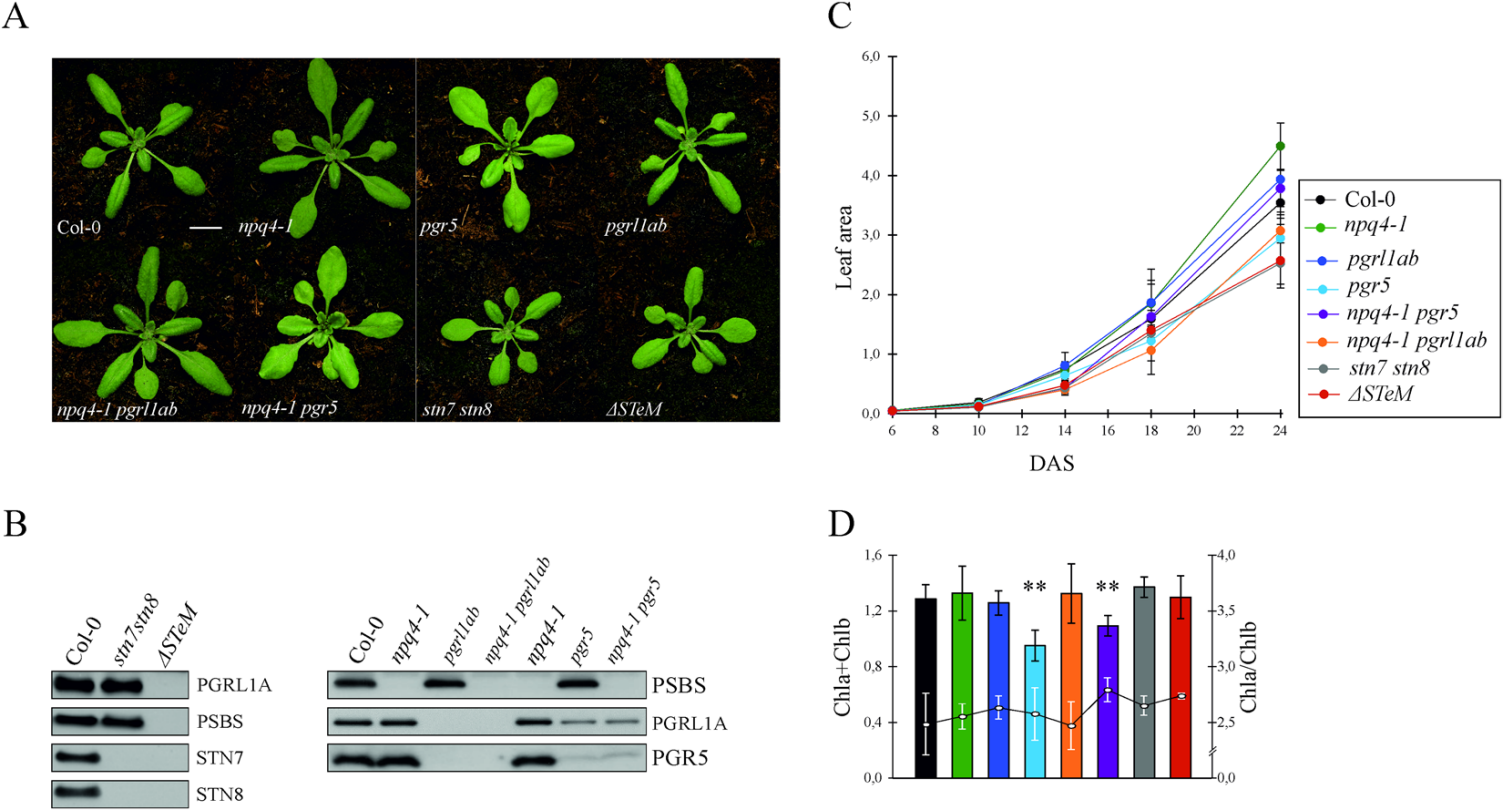
Phenotypes of Col-0 and mutant plants lacking short-term photoprotective mechanism, such as the *npq4-1* mutant lacking the PSBS subunit responsible of NPQ, the *pgrl1ab* and *pgr5* mutants devoid of the PGR5-PGRL1 protein complex that contributes to the formation of the ΔpH transthylakoidal gradient, the *stn7* and *stn8* mutants lacking the thylakoid STN kinases and the sextuple *ΔSTeM* mutant with no short-term regulatory mechanisms. (**A**) Images of Col-0 and mutant plants grown under long-day conditions in a growth chamber for 24 days. The size bar corresponds to 1 cm. (**B**) Immunoblots of fractionated total proteins from Col-0 and mutant leaves probed with antibodies specific for PSBS, PGRL1A, PGR5, STN7 and STN8 proteins. (**C**) Growth rate measurements of plants grown under long-day conditions in a growth chamber for 24 days. Leaf area is expressed as cm^2^ (DAS, Days after sowing). (**D**) Chlorophyll content expressed as μg mg^−1^ leaf fresh weight (histogram) and ratio between Chl a and Chl b (curve). Pigments were extracted from adult plants grown under long-day conditions in a growth chamber for 24 days. Bars indicate the standard deviation and the asterisks represents the statistical significance (**p-value < 0,01), as evaluated by ANOVA test and Student t-test.

**Figure 2:**
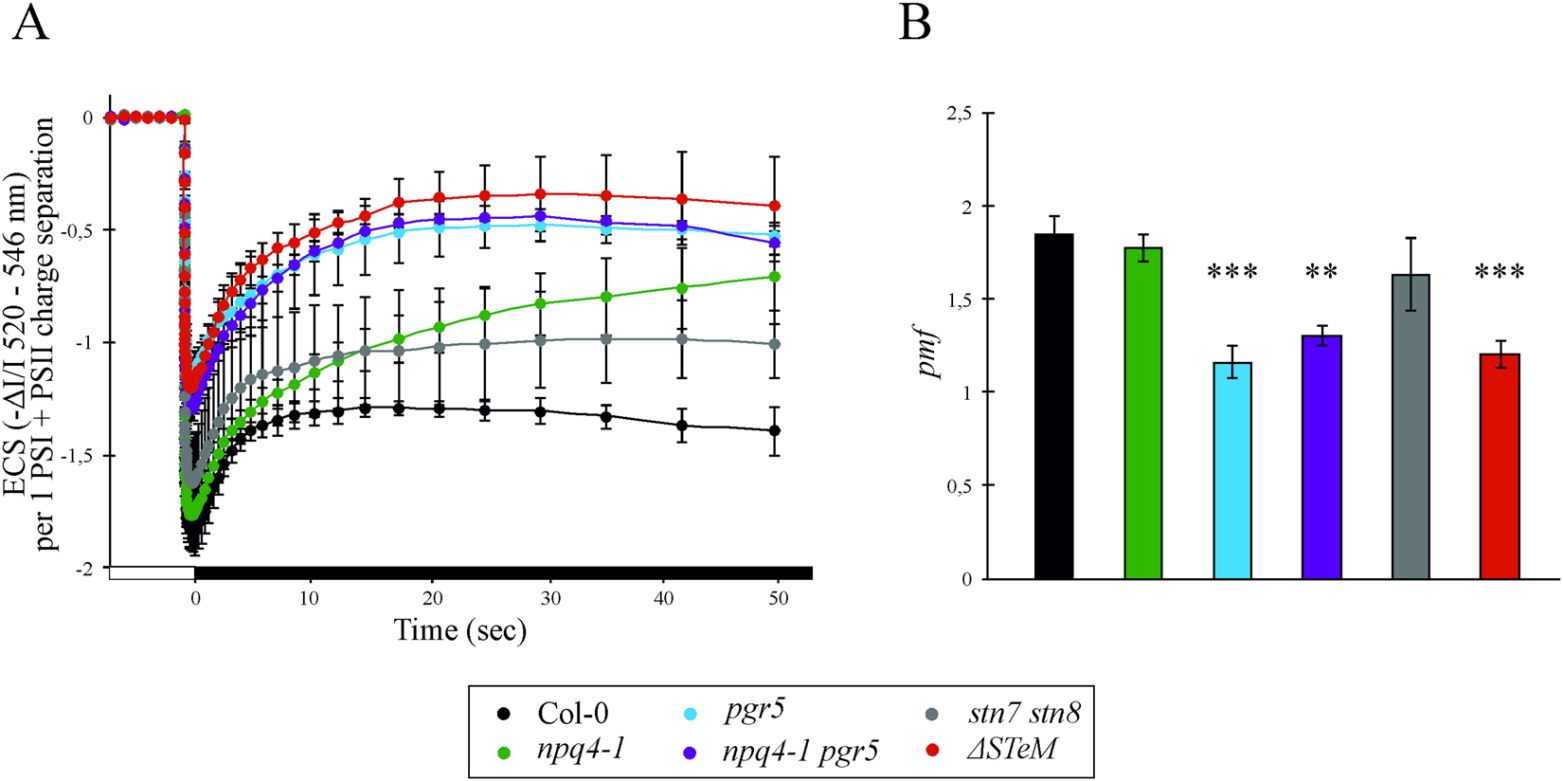
*In vivo* kinetic of ECS dark relaxation, measured in Col-0, *npq4-1*, *pgr5*, *npq4-1 pgr5*, *stn7 stn8* and *ΔSTeM* plants adapted to moderate-light (50 μmol photons·m^−2^·s^−1^). (**A**) Detached leaves were exposed to actinic light (500 μmol photons m^−2^ s^−1^) for 5 min and ECS relaxation was measured during the light‐to‐dark transition (Time 0). White boxes indicate actinic light illumination, while black boxes indicate dark recovery. (**B**) The proton motive force (*pmf*) was calculated, accordingly. Measurements were performed in at least 3 biological replicates, average values and standard deviations are indicated. ECS signals were normalized on PSI + PSII charge separation signals (see Fig. S1C,D). Bars indicate the standard deviation and the asterisks represents the statistical significance (**p-value < 0,01; ***p-value < 0,001) as evaluated by ANOVA test and Student t-test. Note, that we preferred to report the *pmf* rather than ΔpH values, since the real partitioning of the *pmf* between its two components (ΔΨ and ΔpH) is still debated^58,59^.

In order to dissect the interconnections and the relative importance of short-term photoprotective mechanisms, mutations that abolish PSBS-dependent NPQ (*npq4-1*, lacking the PSBS subunit of photosystem II), ΔpH mutants (*pgr5* and *pgrl1a pgrl1b*, henceforth referred to as *pgrl1ab*, devoid of the PGR5-PGRL1 protein complex) and thylakoid protein phosphorylation (*stn7 stn8*, lacking the thylakoid-associated STN kinases) have been combined with the aim to obtain both higher order mutants (*npq4-1 pgrl1ab, npq4-1 pgr5*) and the sextuple mutant, lacking the entire set of short-term regulatory mechanisms, hereafter referred to as *ΔSTeM* (Fig. 1A). Worth to note that about 25% of PGRL1 protein is still detectable in *pgr5* and *npq4-1 pgr5* thylakoids (Fig. 1B), whereas no accumulation of PGR5 protein is observable in *pgrl1ab* and *npq4-1 pgrl1ab* (see also Dal Corso *et al*.^27^). As shown in Fig. 1A,C, a marked reduction in the growth rate is observed in the *stn7 stn8* and in the *ΔSTeM* mutants when grown under optimal conditions (growth light intensity of 100 μmol photons m^−2^ s^−1^, over a photoperiod of 16 h light/8 h dark). In particular, *stn7 stn8* and *ΔSTeM* growth rates appear comparable to Col-0 until 14 days after sowing (DAS), whereas they diverge at 18 DAS resulting in rosettes with a decreased size at 24 DAS (Fig. 1A,C). No major differences are, instead, observed in the total chlorophyll content (Chl a + b) and Chl a/b ratio between the entire set of mutants and Col-0 (Fig. 1D), with the exception of *pgr5* leaves that accumulate less chlorophyll without changing the Chl a/b ratio. To confirm the main role of PGR5-PGRL1 protein complex in the formation of the proton motive force (*pmf*), Col-0, *npq4-1*, *pgr5*, *npq4-1 pgr5*, *stn7 stn8* and *ΔSTeM* plant lines were subjected to the kinetic analysis of the electrochromic pigment absorbance shift (ECS) (Fig. 2). Leaf material adapted to moderate-light (50 μmol photons·m^−2^·s^−1^) was exposed for 5 min to red actinic light (LED, 500 μmol photons m^−2^ s^−1^), then relaxed in the dark for 50 seconds (Fig. 2A), and the ECS relaxation kinetic was measured during the light‐to‐dark transition (Fig. 2B). As described in Fig. 2A,B, *npq4-1* and *stn7 stn8* mutants showed an ECS kinetic similar to Col-0 control, generating a comparable *pmf*. On the contrary, *pgr5*-containing plant lines showed a marked difference in ECS relaxation kinetics, revealing a significantly reduced (~30% drop) capability of *pgr5*, *npq4-1 pgr5* and *ΔSTeM* in generating *pmf*, when acclimated to moderate-light conditions (50 μmol photons m^−2^ s^−1^). These findings are in line to what previously reported^22,23,28^. An identical analysis was performed after 4 h exposure to 500 μmol photons m^−2^ s^−1^ white light, and comparable differences between *pgr5*-containg plants and Col-0 were observed (Fig. S1A,B).

**Figure 3:**
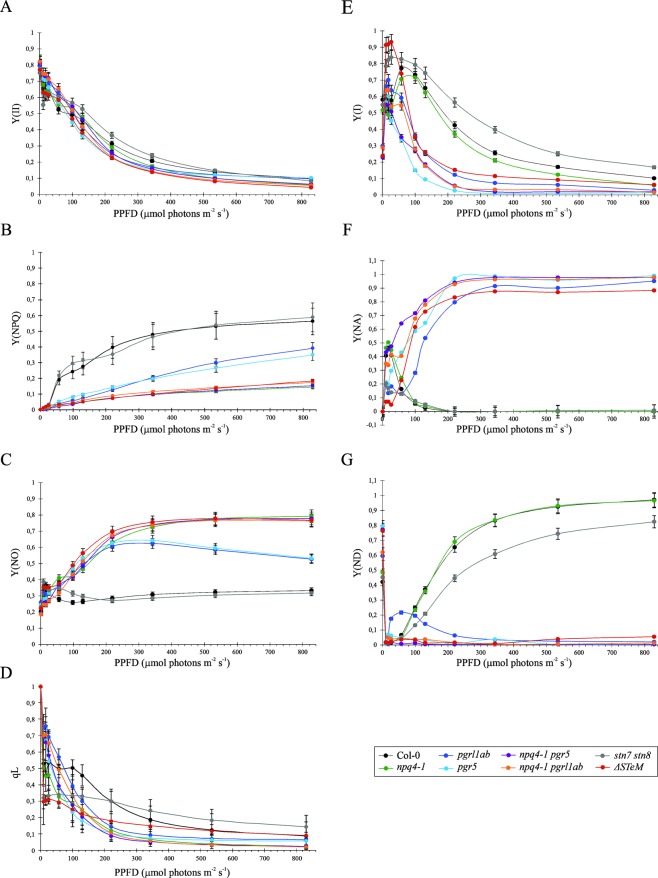
Photosynthetic performance of PSII and PSI complexes in Col-0 and mutant leaves. (**A–D**) Parameters of PSII functionality Y(II), Y(NPQ), Y(NO), and qL were measured after 30 min of dark adaptation and upon exposure to increasing light intensities. (**E–G**) PSI efficiency was also evaluated, through the quantification of Y(I), Y(ND) and Y(NA) parameters; (PPFD, photosynthetic photon flux density).

In addition, the effective quantum yield of PSII [Y(II), see Fig. 3A], measured after dark-adaptation and at increasing actinic light intensities (0 to 829 μmol·photons m^−2^·s^−1^) was generally comparable among the different genotypes, despite the PSBS-dependent NPQ [Y(NPQ)] resulted to be completely abolished in the *npq4-1*-containing genotypes and dramatically decreased in *pgrl1ab* and *pgr5* mutants, in which reached 40% of Col-0 level, after exposure to high light (Fig. 3B). In agreement with these observations, values of the Y(NO) parameter, indicating the energy quenching processes occurring within the PSII reaction center^29,30^, rapidly raised as light intensity increased in the *npq4-1*-containing mutants, whereas ΔpH mutants, showed a peculiar kinetic, characterized by a *npq4*-like behavior, at moderate light intensities, and lower values at higher light intensity (>400 μmol·m^−2^·s^−1^), as the mutants succeeded to establish a proton gradient in the lumen and to induce the PsbS-dependent NPQ (Fig. 3C, see also Tikkanen *et al*.^31^,). In agreement with that, the fraction of open PSII centers^29^ (qL, Fig. 3D) resulted to be higher in Col-0 and *stn7 stn8* leaves than the rest of the genetic backgrounds, particularly at moderate light intensities (100–350 μmol·photons m^−2^·s^−1^).

An identical experimental set-up was used to assess PSI activity in the different genetic backgrounds (Fig. 3E–G). In particular, the quantum yield of PSI, Y(I), was relatively high in Col-0, *npq4-1* and *stn7 stn8* mutant plants even at high light intensities (till around 500 μmol·m^−2^·s^−^1), whereas single and multiple mutants devoid of the PGR5-PGRL1 protein complex showed a marked drop of PSI yield at moderate-to-high light intensities (>100 μmol·photons m^−2^·s^−1^), as a consequence of their inability to efficiently oxidize the P700 chlorophyll pair (Fig. 3E, Tikkanen *et al*.^31^, Grieco *et al*.^32^). As a matter of fact, the kinetic of Y(NA) parameter, *i.e*. the quantum yield of non-photochemical energy dissipation in PSI due to acceptor side limitation (Fig. 3F), was similar in *npq4-1*, *stn7 stn8* and Col-0 leaves, reaching peaks at low light intensities (around 20–40 μmol photons·m^−2^·s^−1^) and showing a strong decrease at higher light conditions, as soon as the photosynthetic control is engaged. On the other hand, PGR-devoid mutants showed a rapid increase of Y(NA) values, reaching a plateau at light intensity values higher than 300 μmol·photons m^−2^·s^−1^, as a consequence of the over-reduction of PSI acceptor side. Similarly, the Y(ND) values, *i.e*. the non-photochemical PSI quantum yield of donor-side limited heat dissipation (Fig. 3G), showed the incapability of PGR-devoid mutants to accumulate P700 in the oxidized form, as previously described^24,33,34^. Overall, our findings highlight the primary importance of proton gradient regulation (PGR)-dependent photosynthesis-control with respect to PSI yield, especially under moderate actinic light intensities.

### Photoinhibition of PSII is phenomenologically linked to the lack of ΔpH-dependent photosynthesis-control and the consequent over-reduction of PSI reaction centers

**Figure 4:**
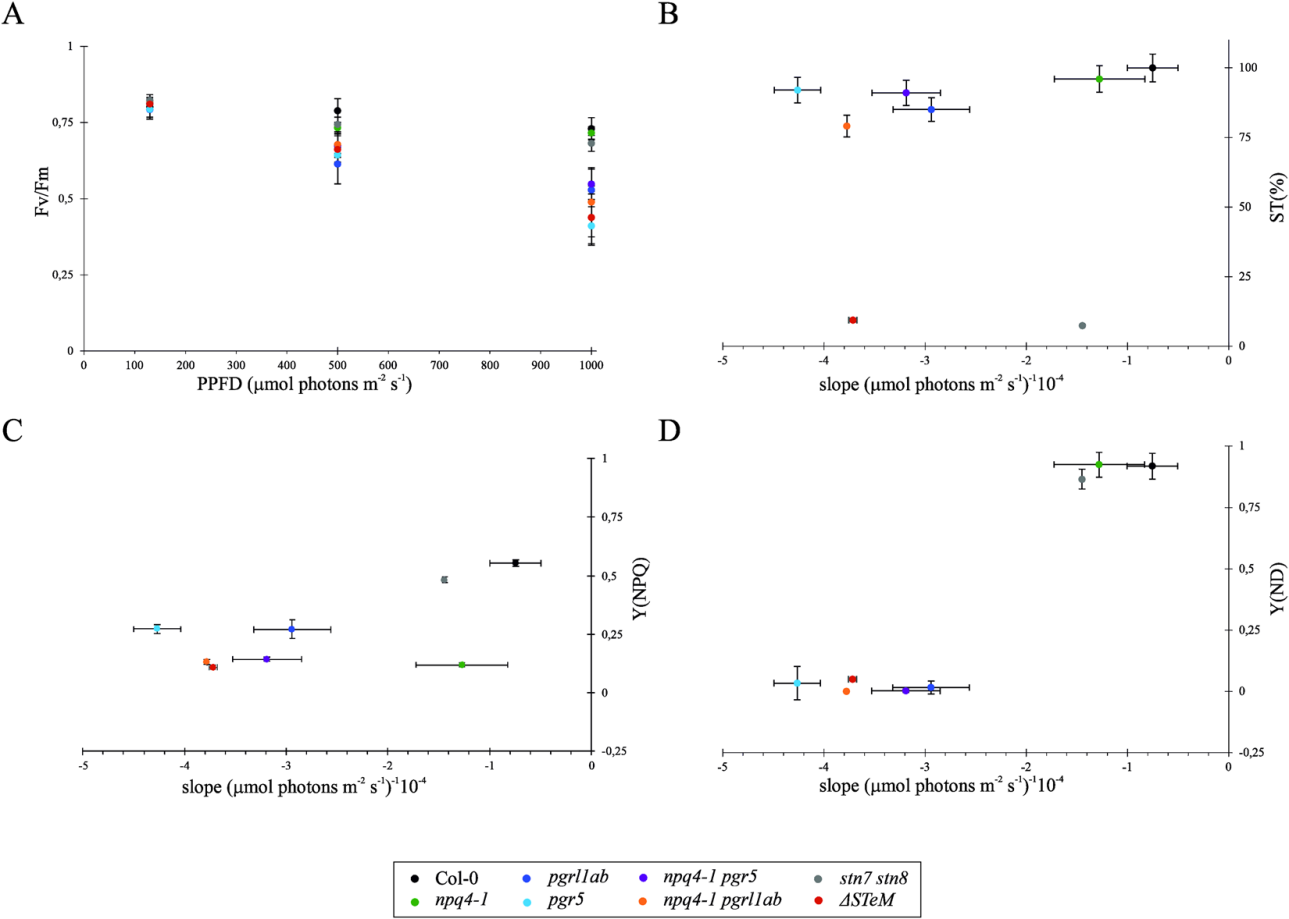
Levels of PSII photoinhibition with respect to the absence of State Transitions (ST), PSBS-dependent NPQ [Y(NPQ)] and ΔpH-dependent photosynthesis-control [Y(ND)]. (**A**) Photoinhibition was estimated by measuring the Y(II) parameter, after 2 h of exposure to 130, 500 and 1000 μmol·photons m^−2^·s^−1^ of actinic light. The slopes of the trend lines in (**A**) were plotted *vs* either state transition percentage (ST%) with respect to Col-0 level (see also Fig. S2) (**B**), Non-Photochemical-Quencing [Y(NPQ)] (**C**), and PSI donor site limitation Y(ND) (**D**). Measurements are shown as average ± s.d. of three biological replicates. Note that NPQ and Y(ND) values refer to Fig. 3B,C, respectively, at 829 μmol·photons m^−2^·s^−1^ of light.

Photoinhibition of Col-0 and mutant plants was evaluated via the maximum quantum yield of PSII (Fv/Fm), measured after 2 hours of exposure to 130, 500 or 1000 μmol·m^−2^·s^−1^ of light (Fig. 4A). The results clearly show that mutants lacking either the STN kinases and, surprisingly, even the PSBS protein behaved very similarly to Col-0. On the contrary, single and higher order mutants devoid of the ability to form a full *pmf* (see also Fig. 2) were strongly photoinhibited, with no major differences between *ΔSTeM* and the *pgrl1ab* and *pgr5* mutants. Thus, it seems that the impairment of ΔpH-dependent photosynthesis-control confers enhanced light sensitivity, irrespectively of whether NPQ or state transitions are developed or not. In particular, when the negative slopes of trend lines obtained from Fig. 4A (used to estimate photoinhibition) were plotted as a function of State Transitions (Figs. 4B and S2; ST%, percentage of state transition with respect to Col-0 values), PSBS-dependent NPQ [Fig. 4C; Y(NPQ)] and Y(ND) (Fig. 4D) values, a clear association with photoinhibition was only displayed by the lines containing the *pgr* mutations. Indeed, *stn7 stn8* and *ΔSTeM* leaves, both devoid of the State Transitions mechanism, had very different slope values: much smaller, therefore indicating higher photoinhibition, in *ΔSTeM* with respect to *stn7 stn8* (Fig. 4B). Similarly, a higher slope value, *i.e*. less photoinhibition, was observed in *npq4-1* leaves with respect to *ΔSTeM* plants lacking both ΔpH-dependent photosynthesis-control and NPQ (Fig. 4C). Thus, taking these findings together, it appears clear that the ΔpH-dependent photosynthesis-control plays a major role in photoprotection.

**Figure 5:**
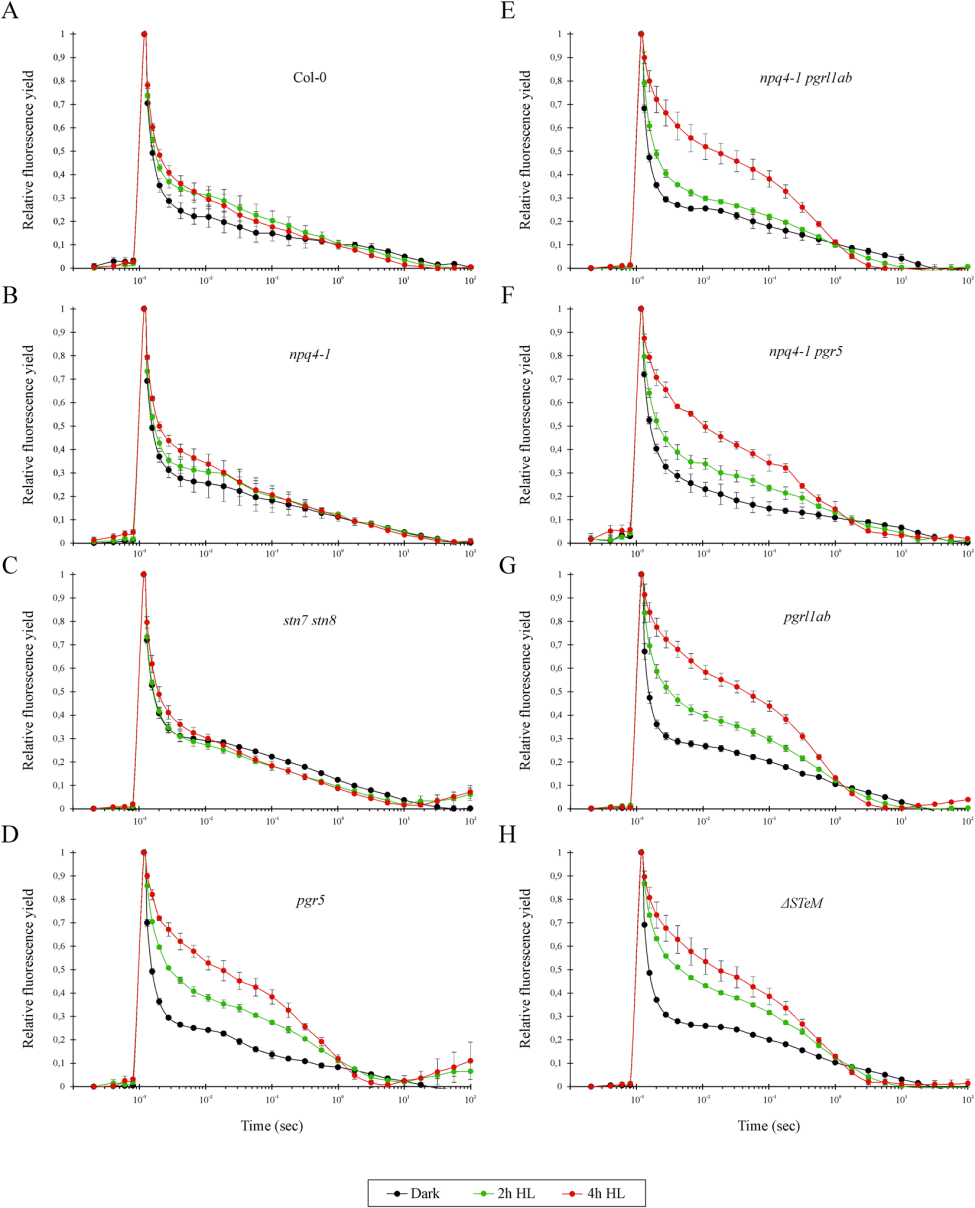
Chlorophyll fluorescence decay, measured in Col-0 (**A**), *npq4-1* (**B**), *stn7 stn8* (**C**), *pgr5* (**D**), *npq4-1 pgrl1ab* (**E**), *npq4-1 pgr5* (**F**), *pgrl1ab* (**G**) and *ΔSTeM* (**H**) leaves after dark adaptation and exposure to HL for 2 and 4 hours. Chlorophyll fluorescence values are reported in the y-axis, where maximum fluorescence was set to 1. The X-axis indicates the decay time scale, from 10^−4^ to 10^2^ sec. Measurements are shown as mean ± s.d., n = 3.

To further characterize the functionality of PSII in the different genetic backgrounds, fluorescence decay measurements in the 10^−4^–10^2^ sec time-range were performed on dark-adapted and HL-treated plants, irradiated with high-light (500 μmol·photons m^−2^·s^−1^) for either 2 or 4 hours (Fig. 5). A single-turnover saturating flash was used to trigger the reduction of Q_A_ with a single electron, extracted from the donor side of PSII, leading to increased fluorescence yield. The subsequent dark-induced re-oxidation of Q_A_^−^ resulted in the relaxation of fluorescence yield and exhibited three main decay phases: fast, middle and slow^35^. For each phase, amplitude and decay time constant were determined, as reported in Table 1. In the case of dark-adapted Col-0 (Fig. 5A), the fast phase, raised from re-oxidation of Q_A_^−^ by plastoquinone bound to Q_B_ site in the dark, contributed to 82% of total amplitude, with a time constant (T_1_) of 309 µs. The middle phase, originated from re-oxidation of Q_A_^−^ by plastoquinone molecules in reaction centers with empty Q_B_ site at the time of the flash light, displayed 9,5% of total amplitude with a time constant (T_2_) of 17 ms. Finally, the slow phase that arises from a back-reaction of the S_2_ state of the water-oxidizing complex with Q_A_^−^, which is populated via the equilibrium between Q_A_^−^Q_B_ and Q_A_Q_B_^−^, had a 8,8% relative amplitude with a time constant (T_3_) of 4.2 sec. Comparable amplitude values for the three phases could also be observed for all the dark-adapted mutant genotypes, although clear differences were present in the time constants of the middle phase, ranging from a minimum value of 13 ms, observed in *npq4-1 pgr5*, to 65 ms calculated for *ΔSTeM*, indicating that under standard growth conditions PSII is working in a similar way in Col-0 and mutant thylakoids. However, when plants were exposed to high light for 2 and 4 hours a totally different scenario appeared. In particular, Col-0 leaves irradiated with high light for 2 or 4 hours decreased the total amplitude of 4 and 11%, respectively, as a result of a reduction of the fast phase and the concomitant increase of the middle and slow phase (Fig. 5A and Table 1). T_1_ and T_2_ remained in the order of 0,3 ms and 15–30 ms, irrespectively of the irradiation length, whereas a marked shortening of T_3_ was observed upon high light exposure. A similar situation was observed in *npq4-1* (Fig. 5B) and *stn7 stn8* (Fig. 5C) leaves, but major differences were detected in mutants defective in ΔpH formation and, therefore, the photosynthesis-control regulatory mechanism (Fig. 5D–H). First of all, a loss of a total amplitude between 25% and 30% is observed in all genotypes after 4 hours of high light exposure, due to a marked loss of the fast phase and the increase of middle and slow phase (Table 1). In addition, while T_1_ remained in the order of 0,3 ms both T_2_ and T_3_ values showed marked drops, further confirming the over-reduction of the electron transport chain in the absence of a normal ΔpH transthylakoidal gradient.

**Table 1:** Chlorophyll fluorescence decay parameters, i.e. amplitude and time constant, measured in Col-0 and mutants after dark adaptation and 2 and 4 hours of high-light (HL) exposure.

	Total amplitude			Fast phase [t_½_(µs)/amp (%)]			Middle phase [t_½_(ms)/amp (%)]			Slow phase [t_½_(s)/amp (%)]		
	Dark	2 h HL	4 h HL	Dark	2 h HL	4 h HL	Dark	2 h HL	4 h HL	Dark	2 h HL	4 h HL
Col-0	100	96	89	309 ± 18^a^/82 ± 3,4^a^	307 ± 18^a^/76 ± 3,3^a^	353 ± 39^a^/70 ± 2,9^a^	17 ± 4^a^/9,5 ± 0,9^a^	29 ± 6^a^/12 ± 0,9^a^	14 ± 2^a^/16 ± 1,1^a^	4,2 ± 1,6^a^/8,8 ± 0,8^a^	1,2 ± 0,3^a^/12 ± 0,9^a^	0,9 ± 0,2^a^/14 ± 0,7^a^
*npq4-1*	100	93	85	283 ± 18^a^/ 81 ± 4,9^a^	313 ± 19^a^/77 ± 3,4^b^	353 ± 21^b^/69 ± 2,5^b^	26 ± 8^A^/8 ± 0,1^A^	37 ± 9^B^/11 ± 1,0^B^	19 ± 3^B^/16 ± 0,9^B^	2,1 ± 0,7^a^/11 ± 0,8^a^	1,8 ± 0,6^b^/12 ± 0,9^b^	1,1 ± 0,2^b^/15 ± 0,7^b^
*pgrl1ab*	100	81	70	258 ± 16^a^/85 ± 4,5^a^	312 ± 25^b^/54 ± 2,0^b^	316 ± 65^b^/31 ± 3,2^c^	51 ± 16^A^/8 ± 0,1^A^	3,7 ± 0,2^B^/17 ± 1,4^B^	6,6 ± 0,3^C^/41 ± 0,3	2,4 ± 0,8^a^/12 ± 0,9^a^	0,43 ± 0,03^b^/29 ± 0,4^b^	0,005 ± 0,003^c^/28 ± 1,9
*pgr5*	100	78	71	301 ± 16^a^/81 ± 3,2^a^	362 ± 66^b^/54 ± 4,1^b^	387 ± 15^c^/38 ± 6,7^c^	36 ± 7^A^/11 ± 0,8^A^	3,3 ± 1,1^B^/20 ± 4,0^B^	5,3 ± 0,8^C^/34 ± 2,6^C^	2,84 ± 1,0^a^/8 ± 0,7^a^	0,28 ± 0,04^b^/26 ± 1,0^b^	0,005 ± 0,003^c^/32 ± 5,4^c^
*npq4-1 pgr11ab*	100	87	74	277 ± 16^a^/82 ± 3,8^a^	300 ± 25^b^/65 ± 2,6^b^	330 ± 54^c^/37 ± 2,8^c^	50 ± 15^A^/8 ± 1,3^A^	3,4 ± 0,7^B^/15 ± 1,8^B^	3,9 ± 0,7^B^/20 ± 2,0^B^	2,97 ± 1,06^a^/10 ± 0,1^a^	0,50 ± 0,05^b^/20 ± 0,1^b^	0,33 ± 0,02^b^/43 ± 0,1^c^
*npq4-1 pgr5*	100	89	77	303 ± 23^a^/78 ± 4,0^a^	327 ± 36^a^/64 ± 3,5^b^	363 ± ^4b9^/41 ± 2,8^c^	13 ± 2,8^A^/13 ± 1,1^A^	5,0 ± 1,2^B^/16 ± 2,0^A^	6,6 ± 1,0^B^/22 ± 1,5^B^	4,81 ± 1,89^a^/9 ± 0,1^a^	0,62 ± 0,01^b^/20 ± 0,1^b^	0,37 ± 0,03^c^/37 ± 0,1^c^
*stn7 stn8*	100	98	92	307 ± 20^a^/78 ± 3,6^a^	246 ± 27^a^/73 ± 4,7^a^	342 ± 40^a^/68 ± 3,^a^9	27 ± 2,8^A^/7 ± 1,0^A^	2,8 ± 0,9^B^/13 ± 2,5^B^	5,4 ± 1,9^B^/14 ± 2,3^B^	1,30 ± 0,33^a^/15 ± 3,2^a^	0,24 ± 0,05^b^/15 ± 3,1^a^	0,22 ± 0,06^b^/18 ± 1,3^a^
*ΔSTeM*	100	78	75	292 ± 18^a^/81 ± 4,1^a^	387 ± 31^b^/51 ± 1,8^b^	332 ± 64^c^/37 ± 3,5^c^	65 ± 2,6^A^/7 ± 1,2^A^	5,5 ± 0,8^B^/17 ± 1,3^B^	5,0 ± 0,1^B^/20 ± 2,1^B^	2,19 ± 0,87^a^/12 ± 1,2^a^	0,44 ± 0,03^b^/32 ± 5,3^b^	0,33 ± 0,03^b^/43 ± 0,1^b^

The time constants of fluorescence decay, indicated as t_½_, and related to fast, middle and slow phases, are reported. The total amplitude of the fluorescence decay, measured in dark-adapted samples, was set to 100. The amplitude of fluorescence decay kinetic for each phase is indicated as percentage of total amplitude. Measurements were performed in triplicates, average values ± s.d. are indicated. Data are grouped in three blocks (i.e. Fast phase, Middle phase, Slow phase). In each group, identical letters mean that there are not significant differences at the level of p-value < 0,05, as evaluated by ANOVA test.

### Thylakoid protein phosphorylation does not have a major impact on D1 protein turnover and PSII photoinhibition

**Figure 6:**
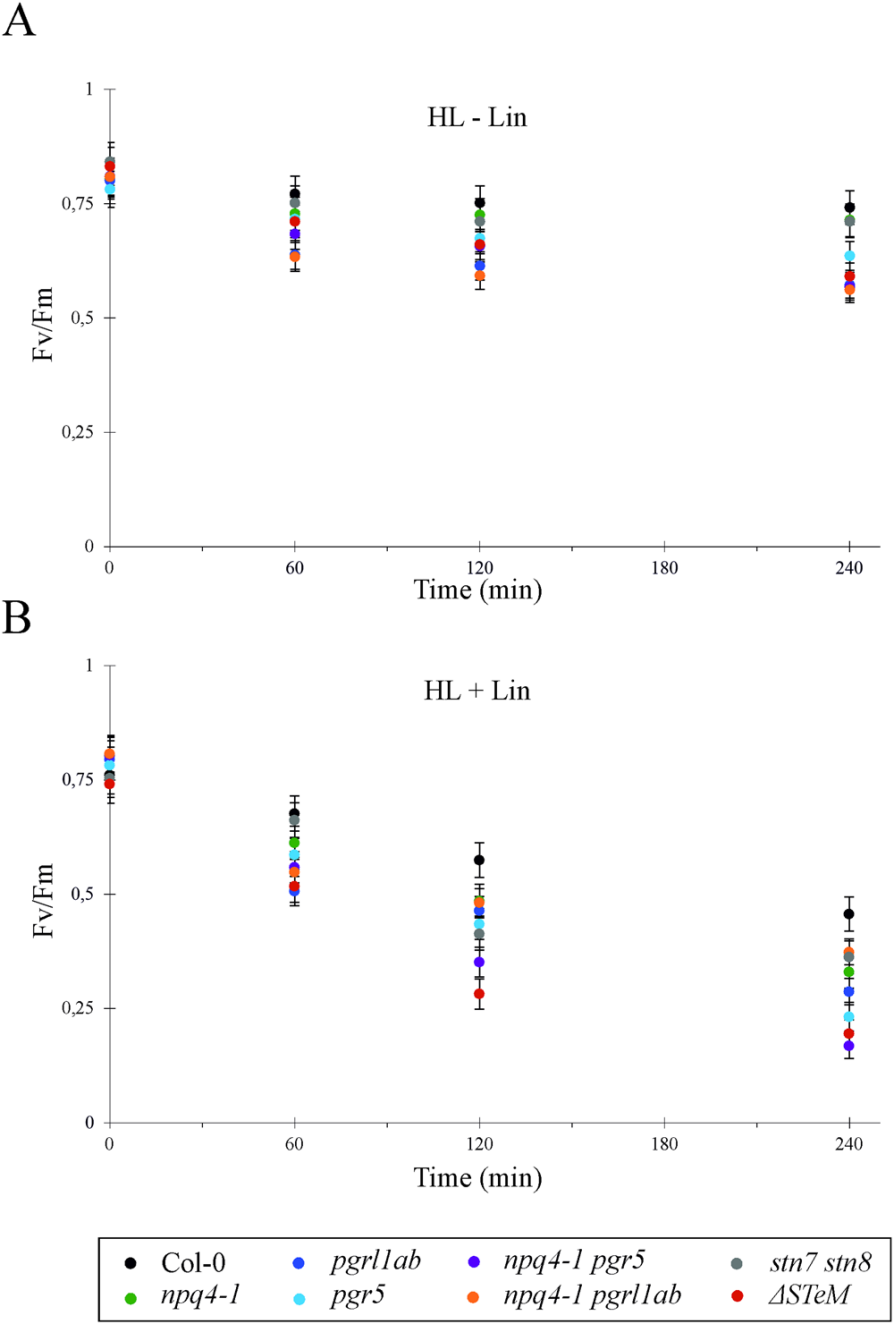
Photoinhibition of PSII estimated as Y(II) decrease, upon irradiation of dark-adapted leaves (values corresponding to 0 min) with high-light (60, 120 and 240 min of 500 μmol photons·m^−2^·s^−1^), in absence (**A**) or presence (**B**) of Lincomycin, measured in Col-0 and mutants. Measurements are shown as mean ± s.d., n = 3.

The level of PSII photoinhibition was also measured as the maximum PSII quantum yield (Fv/Fm), in dark-adapted leaves and in leaves exposed to either optimal growth light (GL, 100 μmol photons m^−2^ s^−1^) or stressful high light (HL, 500 μmol photons m^−2^ s^−1^) for 60, 120 and 240 minutes, in absence or presence of Lincomycin (Lin) (Fig. 6). Under GL condition, where there is no effect of light on Fv/Fm, the addition of Lin leads to a partial loss of PSII activity, linked to the inhibition of the *de novo* synthesis of D1 and, more in general, of plastid-encoded proteins (not shown). Under HL conditions, in the absence of Lin, a general decrease of Fv/Fm values after 60 minutes of HL exposure could be appreciated, more obvious in *pgrl1ab* and *npq4-1 pgrl1ab* mutants (Fig. 6A). A similar trend was observed after 120 minutes of HL treatment, whereas the exposure to HL for 240 min indicated that photosynthesis-control devoid mutants are more susceptible to photoinhibition than Col-0, *stn7 stn8* and *npq4-1*, confirming the data reported in Fig. 4A. As expected, in presence of Lin, all genotypes displayed a similar trend as in GL but with a much larger susceptibility to the HL treatment, as revealed by the considerable decrease of Fv/Fm after 120 and 240 min of HL exposure (Fig. 6B). In particular, Col-0 Fv/Fm was reduced of about 40% after HL treatment for 240 min in presence of Lin, whereas *npq4-1 pgr5* and *ΔSTeM* mutants showed the largest PSII photoinhibition, with Fv/Fm values reduced by 80 and 75%, respectively, in comparison to dark-adapted samples.

**Figure 7:**
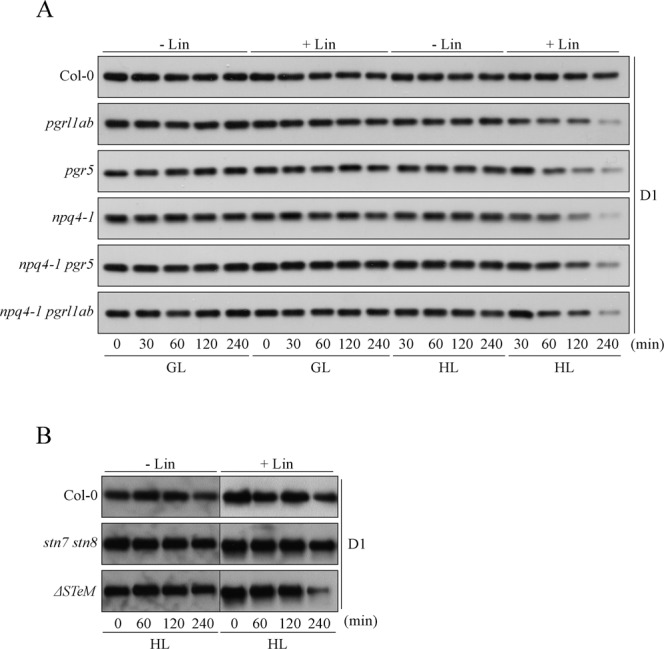
Immunoblot analyses of PSII integrity in Col-0 and mutants impaired in NPQ, ΔpH-dependent photosynthesis-control and State Transitions mechanisms. (**A**) Col-0, *npq4-1*, *pgrl1ab*, *pgr5*, *npq4-1 pgrl1ab* and *npq4-1 pgr5* mutants were dark-adapted in absence or presence of Lincomycin (0 min, +/− Lin) and then exposed to growth-light (GL) and high-light (HL) for 30, 60, 120 and 240 min. The accumulation of D1 subunit of PSII was investigated via immunoblot. (**B**) D1 accumulation was tested in Col-0, *stn7 stn8* and *ΔSTeM* mutants after dark-adaptation (0 min, +/− Lin) and upon 60, 120 and 240 min HL exposure, in absence or presence of Lincomycin.

In addition to PSII photoinhibition evaluated by fluorescence-based methods (PAM, single turnover flash), the same samples were also analyzed for the ability to accumulate D1 protein by immunoblot analyses under GL and HL conditions with and without Lin treatments (Fig. 7). In all tested genotypes, D1 accumulation was not affected under GL conditions in the absence of Lin treatment, whereas leaves incubated overnight with 2,3 mM of Lin and then exposed to GL for 240 min, showed decreased D1 amount similar to the levels observed after HL exposure for 240 min in the absence of Lin. However, when the HL exposure was combined with the Lin treatment, differences became evident. In particular, D1 accumulation was markedly decrease in *npq4-1*, *pgr5*, *pgrl1ab*, *npq4-1 pgrl1ab, npq4-1 pgr5* and *ΔSTeM* with respect to Col-0 amount. Interestingly, no additive effects were observed when the accumulation of D1 protein in *npq4-1* thylakoids was compared with *npq4-1 pgrrl1ab* and *npq4-1 pgr5* and the *ΔSTeM* sextuple mutant, in agreement with the Fv/Fm values reported in Figs. 4A and 6B. On the contrary, Col-0 and the *stn7 stn8* mutant suffered a comparable and marginal decrease of D1 amount after 240 min of HL.

**Figure 8:**
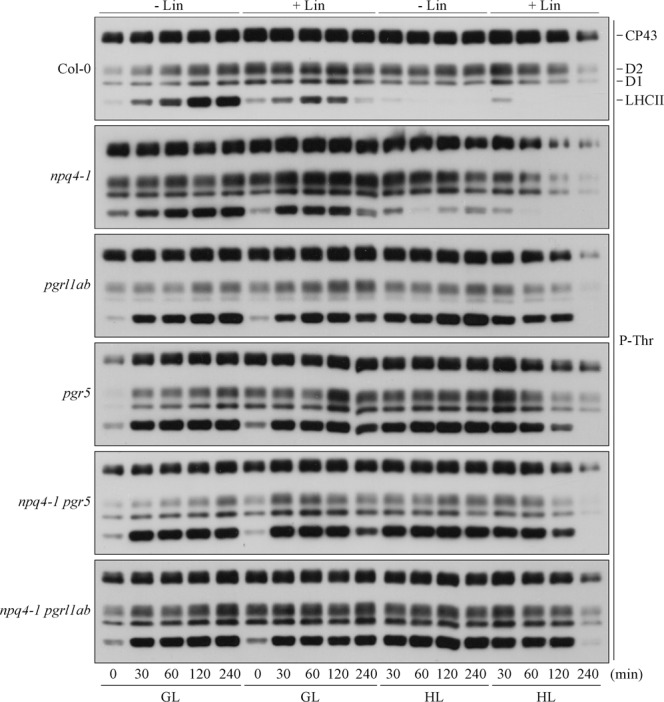
Thylakoid protein phosphorylation pattern. Thylakoid membranes were isolated from Col-0, single and multiple mutants, fractionated onto SDS-PAGE, transferred onto nitrocellulose membranes and probed with a polyclonal anti-phosphothreonine antibody. Levels of phosphorylation of CP43, D2, D1 and LHCII are shown over time (0-to-240 min) upon exposure to optimal growth light (GL) and high-light (HL) conditions. Lincomycin treatment was performed overnight in the dark where indicated (+Lin). One representative immunoblot (n = 3) for each genotype is shown.

The fact that Col-0 and the *stn7 stn8* double mutant do not show major differences with respect to PSII yield under HL stress conditions with and without Lin, points to a marginal role of thylakoid protein phosphorylation with respect to PSII photoprotection. To investigate further this aspect, the thylakoid phosphorylation pattern was monitored in plants devoid of either NPQ or ΔpH-dependent photosynthesis-control and in the corresponding mutants where both mechanisms are inactivated (Fig. 8). In agreement with previous observations, the exposure of Col-0 leaves to GL led to a general increase in phosphorylation of all main phosphoproteins, *i.e*. LHCII, D1 and D2, over time (0-to-240 min), whereas CP43 was already strongly phosphorylated in our experimental conditions. The addition of Lin increased the phosphorylation level of PSII-core proteins even in the absence of light and a comparable accumulation of PSII-core phosphoproteins was maintained until 120 min of GL exposure. On the contrary, P-LHCII signal reached its peak at 60 min, markedly decreased at 120 min and disappear after 240 min of GL with Lin treatment. HL exposure in the absence of Lin maintained a relatively high accumulation of CP43, D1 and D2 phosphoproteins, comparable to what observed at 120–240 min of GL conditions, throughout the tested time points. However, LHCII phosphorylation was barely detectable after 30 min and disappeared after 60 min exposure to high light. The addition of Lin to HL conditions resulted in a gradual loss of phosphorylation levels. In general, the PSII-core phosphorylation pattern observed in mutant plants (see Fig. 8) was very similar to Col-0 under the different light regimes in presence or absence of Lin, although the accumulation of PSII core phosphoproteins was markedly higher in *npq4-1* thylakoids and clearly reduced in *pgrl1ab* in comparison to Col-0. Notably, the D1 phosphoprotein was barely detectable in *pgrl1ab* thylakoids even after 240 min of GL exposure. On the contrary, the LHCII phosphorylation pattern, upon HL illumination, was markedly different between Col-0 and mutant plants devoid PGR proteins. In particular, LHCII phosphorylation was retained in *pgr* mutants upon high light treatment, unlike Col-0 and *npq4-1* thylakoids where high light exposure suppressed LHCII phosphorylation. This phosphorylation pattern resembles the one of *tap38* mutant^36^ and is certainly the consequence of the high reduction state of the thylakoid electron transport carriers, including Cyt *b*_6_*f*, upon depletion of the ΔpH-dependent photosynthesis control.

### PSII photoinhibition guarantees PSI integrity

In order to evaluate the impact of PSII photoinhibition on PSI integrity, Fv/Fm and Pm (the maximal change of the P700 signal upon quantitative transformation of P700 from the fully reduced to the fully oxidized state) parameters were measured from dark-adapted and HL-treated (2 and 4 hours) plants, in either absence or presence of Lin (Fig. S3 and Table 2). In the dark, Fv/Fm did not shown any marked difference among genotypes (p < 0,05), whereas the Pm parameter was higher in Col-0, *npq4-1*, *stn7 stn8*, than all *pgr*-containing mutants (p < 0,05). The Fv/Fm values slightly decreased by increasing the length of exposure to HL, while addition of Lin led to a marked drop of Fv/Fm values (see Table 2). On the other hand, Pm values remained higher than 0,40 at the different HL regimes, irrespectively of the presence or absence of Lin. On the contrary, PGR-devoid mutants were highly sensitive to high light conditions, displaying Fv/Fm values in the range of 0,65–0,31, much lower than 0,78 observed in Col-0 (Fig. S3 and Table 2). In addition, PSI activity was found to be lower than 0,15 in *pgr5*, *npq4-1 pgrl1ab* and *npq4-1 pgr5* thylakoids under the same conditions. Interestingly, the addition of Lin to the high-light treatment restored PSI activity in PGR-devoid mutants, while PSII efficiency dropped to values even lower than 0,30, as in the case of *pgr5*, *npq4-1 pgr5* and *ΔSTeM* leaves. Overall, these findings indicate that in the absence of the PGR-dependent photosynthesis control, a marked inhibition of PSII activity is beneficial to prevent PSI inactivation, highlighting further the primary importance of photosynthesis control in photoprotection of PSI.

**Table 2:** Photosynthetic efficiency of PSII and PSI measured in Col-0 and mutants after dark adaptation and exposure for 2 and 4 hours to high-light (HL) in either absence or presence of Lincomycin.

	Dark		2 h HL		4 h HL		2 h HL + Lin		4 h HL + Lin	
	F_V_/F_M_	Pm	F_V_/F_M_	Pm	F_V_/F_M_	Pm	F_V_/F_M_	Pm	F_V_/F_M_	Pm
Col-0	0,814 ± 0,046^a^	0,505 ± 0,024^A^	0,799 ± 0,007^a^	0,527 ± 0,011^B^	0,785 ± 0,004^b^	0,517 ± 0,011^A^	0,561 ± 0,025^c^	0,512 ± 0,037^A^	0,490 ± 0,016^d^	0,536 ± 0,004^A^
*npq4-1*	0,802 ± 0,00697^a^	0,87 ± 0,00697^A^	0,758 ± 0,0194^a,b^	0,83 ± 0,05^A^	0,722 ± 0,0136^b^	0,83 ± 0,05^A^	0,450 ± 0,021^c^	0,87 ± 0,03^A^	0,401 ± 0,019^c^	0,83 ± 0,03^A^
*pgrl1ab*	0,807 ± 0,006^a^	0,285 ± 0,023^A^	0,677 ± 0,020^b^	0,197 ± 0,012^A^	0,650 ± 0,016^b^	0,151 ± 0,007^B^	0, 496 ± 0,027^b^	0,286 ± 0,053^A^	0,293 ± 0,036^c^	0,286 ± 0,023^A^
*pgr5*	0,794 ± 0,005^a^	0,259 ± 0,020^A^	0,628 ± 0,022^b^	0,107 ± 0,01^B^	0,597 ± 0,033^c^	0,037 ± 0025^B^	0,521 ± 0,018^d^	0,241 ± 0,035^A^	0,223 ± 0,005^e^	0,215 ± 0,042^A^
*npq4-1 pgr11ab*	0,806 ± 0,002^a^	0,234 ± 0,027^A^	0,535 ± 0,048^b^	0,187 ± 0,011^A^	0,320 ± 0,038^c^	0,063 ± 0,053^B^	0,457 ± 0,022^b^	0,287 ± 0,027^A^	0,330 ± 0,050^b^	0,266 ± 0,030^A^
*npq4-1 pgr5*	0,811 ± 0,006^a^	0,382 ± 0,022^A^	0,625 ± 0,023^b^	0,126 ± 0,006^B^	0,467 ± 0,023^c^	0,025 ± 0,017^C^	0,451 ± 0,020^c^	0,197 ± 0,008^B^	0,226 ± 0,035^d^	0,117 ± 0,034^B^
*stn7 stn8*	0,807 ± 0,008^a^	0,410 ± 0,014^A^	0,724 ± 0,005^a^	0,437 ± 0,055^A^	0,685 ± 0,065^a^	0,438 ± 0,019^A^	0,561 ± 0,060^b^	0,468 ± 0,044^A^	0,525 ± 0,013^b^	0,406 ± 0,041^A^
*ΔSTeM*	0,811 ± 0,004^a^	0,268 ± 0,011^A^	0,444 ± 0,063^b^	0,084 ± 0,021^B^	0,314 ± 0,015^b^	0,031 ± 0,011^B^	0,408 ± 0,023^b^	0,261 ± 0,040^A^	0,294 ± 0,013^b^	0,217 ± 0,018^A^

Chl fluorescence emission and P700^+^ absorbance were recorded to monitor PSII and PSI activity, respectively. Photosynthetic efficiency related to PSII and PSI (F_V_/F_M_ and Pm), respectively, were calculated as reported in Materials and Methods. Measurements were performed at least in triplicates, average values ± s.d. are indicated. Lower case letters are referred to Fv/Fm, whereas upper case letters are referred to Pm. Same letter mean no significant differences at the level of *p-value* < 0,05, as detected by ANOVA test.

## Discussion

Light induced inactivation of PSII causes enhanced degradation of the D1 protein, while the PSII recovery relies on *de novo* synthesis of D1. Under PSII photoinhibitory conditions (high light), activity and stability of PSI is not affected, unless high light treatment is performed in cold environment^10^ or in mutant backgrounds lacking the PGR5/PGRL1 complex, in which the ability to form a normal ΔpH and activate the photosynthesis-control is not working properly^11,12^.

A large number of molecular processes have been suggested to function as protection mechanisms against an excess of light. Among those, the most relevant consists in the formation of the PSBS-dependent component of NPQ. Nevertheless, several authors argued that NPQ could have only a little role in the direct photoprotection of PSII, while could be important for the PSII recovery^14^. Our data from *PSBS*-depleted mutants are in line with these findings, as *npq4-1* plants show sensitivity to high light similar to wild type. Accordingly, in a very recent study is reported that *npq4-1* mutant, after 10 h irradiation with 1500 μmol photons m^−2^ s^−1^ showed a Fv/Fm ratio of about 0.48–0.50 whereas for the wild type the ratio was about 0.58–0.60^37^. However, the fact that high light irradiation, combined with Lincomycin treatment, led to the enhanced degradation of D1 in *npq4-1* mutant, indicated that, in mutant background devoid of PSBS, the turnover of D1 is constitutively higher. A similar effect on D1 turnover in PSBS-less mutant was previously reported^38^. Thus, the loss of PSII activity is not observed as long as the rate of damage does not exceed the rate of repair^5,6^, indicating that the absence of PSBS-dependent NPQ is compensated by up-regulation of PSII repair. From these observations, we can suggest that the ability to engage a full NPQ might actually act as a signal aimed to regulate the D1 turnover, as well as a direct photoprotection mechanism meant to prevent D1 degradation. From a redox point of view, a reduced level of NPQ correlates with a higher accumulation of centers with a reduced Q_A_. As Q_A_^−^Q_B_ is in equilibrium with Q_A_Q_B_^−^, it could be expected that in PSBS-less mutants a higher fraction of centers could accumulate a semi-reduced secondary quinone acceptor, which, according to previous works^39,40^, could play a role as a photosensitizer for enhanced degradation of the D1 protein. This could be the mechanism by which the turnover of D1 protein in the *npq4-1* mutant is constitutively higher with respect to the wild-type, although it has to be taken into account that the high turnover rate of D1 and the accumulation of reduced Q_A_ is not usually associated with reduced NPQ in wild-type leaves under physiological conditions.

Furthermore, it should be noted that Y(NO) reflects the energy quenching processes occurring within PSII reaction center with Q_A_ in a reduced state, and that the reduction of Q_A_ has been suggested to be a major requirement and a prerequisite for an efficient PSII reaction centre quenching^41–43^. Therefore, the substantial increase of Y(NO) observed in the *npq4-1*-containing mutants, and in *pgr* mutants at moderate light intensities (see Fig. 3), also suggest the activation of PSII reaction center quenching, as a compensatory mechanism for an effective photoprotection, although this aspect is still debated.

In addition to that, mutants with defect in building up proper trans-thylakoidal pH gradient, such as *pgr5* and *pgrl1ab*, show enhanced degradation of D1, similarly to PSBS-less mutant. In particular, in PGR-devoid mutants, the treatment with high light caused a strong inactivation of PSII, even in the absence of lincomycin. As they are able to engage about 40% of the NPQ observed in wild type and are much more sensitive to light than PSBS-less plants (where the extent of NPQ is near zero), we conclude that, at least in our experimental conditions, the PGR-dependent photosynthesis-control act as an efficient photoprotection mechanism.

Furthermore, unlike wild type and *npq4-1* plants, the photosynthesis-control depleted mutants are not able to photo-accumulate P700^+^, as their Y(ND) is near zero at any light intensity due to the low values of both thylakoid proton gradient (ΔpH) and proton motive force (*pmf*) they can develop^44^. At the same time, they are characterized by the overreduction of PSI acceptors, observed as an increase of Y(NA). Thus besides PSII, PSI is also photodamaged in these mutants, likely because of impairment of iron-sulfur clusters^13^. As no additive phenotypic effects are observed between the photosynthetic characteristics of ΔpH mutants (*pgr5* and *pgrl1ab*) and the ones of higher order mutants *(npq4-1 pgr5, npq4-1 pgrl1ab*, *ΔSTeM*), it can be concluded that the short-term light adaptation is highly depending on the photosynthesis-control regulatory mechanism. Accordingly, mutants lacking of NDH-dependent CET such as *crr2-2, crr-3, crr4-2*^23^ but still able to photo-accumulate P700^+^, are more light resistant than the *pgr5* mutant, deficient in CET and unable to photoaccumulate P700 in the oxidized form^25^.

It is noteworthy that PSI photodamage in PGR-depleted mutants can be markedly reduced through the inhibition of PSII activity, as a consequence of the fact that the amount of electrons injected in the intersystem transport chain is decreased. This indicates that the photosynthesis-control is the main regulator of photosynthetic electron transport and that PSII photoinhibition is the very last option to reduce PSI photodamage^15^. On the other hand, damages to PSI are relevant in inducing inactivation of PSII: the acceptor side of PSII becomes over-reduced and this, in turn, increases the rate of charge recombination with formation of ^3^P680^35^ and PSII inactivation. In addition, the absence of ΔpH-dependent photosynthesis-control affects the value of pmf^44^, and this could alter the electron transfer between Q_A_ and Q_B_, promoting PSII inactivation.

Overall, it appears clear that the ΔpH-dependent photosynthesis control is essential for safeguarding the entire photosynthetic electron transport chain in the thylakoid membrane, and its failure induces a rapid and coordinated inactivation of both PSII and PSI. ΔpH-dependent photosynthesis-control thus maintains the optimal balance between the two main power-units of the photosynthetic apparatus.

## Methods

### Plant material and growth conditions

*Arabidopsis thaliana* single mutant lines were obtained from the Arabidopsis Stock Center (http://arabidopsis.info/). The *npq4-1* mutant was described in Li *et al*.^17^, the *pgrl1a pgrl1b* (also referred as *pgrl1ab*) double mutant in Dal Corso *et al*.^27^, *pgr5* in Munekage *et al*.^22^ and the *stn7 stn8* double mutant in Bonardi *et al*.^19^. All mutant lines are in the Columbia-0 (Col-0) genetic background, except for *pgr5* that has been isolated in Columbia *gl1*. The multiple mutants were obtained by manual crossing and PCR-based segregation analyses. Arabidopsis plants were grown under controlled growth-chamber conditions as described previously^45^, with a growth light intensity of 100 μmol photons m^−2^ s^−1^, over a photoperiod of 16 h light/8 h dark. High light treatment, otherwise stated, was performed with a light intensity of 500 μmol photons m^−2^ s^−1^ for the indicated duration. Lincomycin treatment was performed by floating detached leaves overnight in a solution of lincomycin (1 mg ml^−1^ corresponding to a concentration of 2, 3 mM) in water.

### Chlorophyll quantification and growth rate measurements

For chlorophyll quantification, 50 mg of leaves from 4-week-old Arabidopsis plants were ground in liquid nitrogen. Ground material was resuspended in 90% acetone and centrifuged ay 16.000 *g* for 5 min. Chl a and b concentrations were measured according to Porra *et al*.^46^. Chlorophyll measurements were performed in triplicate. Growth rate was determined by evaluating the leaf area with ImageJ 1.43 u (http://imagej.nih.gov/ij/index.html) software. 12 plants per genotype were measured.

### Immunoblot analyses

Thylakoid isolation was performed as reported^47^ from 4-week-old plants. For immunoblot analyses, thylakoid proteins, corresponding to 3 μg of chlorophyll, were prepared as described^48^ and fractionated by SDS–PAGE (12% w/v acrylamide^49^). Proteins were then transferred onto polyvinylidene difluoride membranes^50^, and replicate filters were immunodecorated using specific antibodies. The PGR5-specific antibody was obtained from Toshiharu Shikanai, Phospho-Threonine Antibody from Cell Signaling Technology, PsbS from Agrisera whereas D1^51^, PGRL1A^27^, STN7 and STN8^52^ antibodies were produced in our laboratory.

### Chlorophyll a fluorescence

*In vivo* chlorophyll a fluorescence and P700 absorbance were measured at different light intensities using the Dual-PAM 100 (Walz, http://www.walz.com/) as previously described^45^.The PSII Fv/Fm, Y(II), Y(NO), Y(NPQ) parameters, together with PSI yield [Y(I)], Pm, donor side Y(ND) and acceptor side Y(NA) limitations, were calculated as reported^53,54^. In the case of Fv/Fm parameter, measurements were performed after 20 min of dark adaptation. State transitions measurements were performed as previously described^55^. In particular, state transitions were monitored on detached leaves with the DualPAM-100 fluorometer. Leaves were exposed to a 800 ms flash of saturating white light to determine *F*_m_, and subsequently illuminated for 15 min with 25 µmol photons m^−2^ s^−1^ red light (PSII light) directly in the PAM fluorometer. Far-red (PSI) light (intensity step 15) was turned on, and after 15 min the maximum fluorescence yield in state 1 (*F*_m1_) was determined. The far-red light was then switched off and the fluorescence recorded for 15 min, after which the maximum fluorescence yield in state 2 (*F*_m2_) was determined. The relative change in fluorescence was calculated as *F*_r_ = [(*F*_i′_ - *F*_i_) − (*F*_ii′_ - *F*_ii_)]/(*F*_i′_ - *F*_i_), where F_i_ and F_ii_ designate fluorescence in the presence of PSI light in state 1 and state 2, respectively, while F_i′_ and F_ii′_ designate fluorescence in the absence of PSI light in state 1 and state 2, respectively. Decay of flash-induced chlorophyll fluorescence was measured by the double modulation fluorometer FL-3500 (PSI, Brno, Czech Republic) and data were analyzed as described^35^. Multicomponent deconvolution of the measured curves was performed by using a fitting function with two exponential components and one hyperbolic component: *F*_*(t)*_ *– F*_0_ = *A*_1_exp(*−t*/T_1_) + *A2*exp(−*t*/T_2_) + *A*_3_/(1 + *t*/T_3_) + *A*_0_. F_(t)_ is the variable fluorescence yield, F_0_ is the basic fluorescence before the flash, A_0_ to A_3_ are the amplitudes and T_1_ to T_3_ are the time constants. Very slowly decaying fluorescence is described by a constant A_0_ amplitude. All measurements were performed after 20 min dark adaptation.

### Electrochromic pigment absorbance shift measurements

*In vivo* electrochromic pigment absorbance shift (ECS) analyses were performed with a JTS-10 spectrophotometer (Biologic, France) on detached leaves, adapted to moderate-light (50 μmol photons·m^−2^·s^−1^) or treated with high-light (500 μmol photons·m^−2^·s^−1^) for 240 min. Leaf material was exposed to red actinic light (500 μmol photons m^−2^ s^−1^) for 5 min and ECS relaxation was measured during the light‐to‐dark transition. Data were collected as the difference between the signals at 520 and 546 nm as described by Cruz *et al*.^56^ and Avenson *et al*.^57^. The amplitude of the ECS signal was normalized to the signal corresponding to one PSI + PSII charge separation, calculated after the application of xenon-induced ECS signals. The *pmf* was evaluated following ECS relaxation kinetics after actinic light switch off.

### Data analysis

Data (statistics analysis, data fitting) were analysed by using the software package OriginPro 9.0 (Microcal SR2; Northampton MA01060 USA).

## Supplementary information

Supplementary Information.
