# Supplementary material for: Higher order photoprotection mutants reveal the importance of ΔpH-dependent photosynthesis-control in preventing light induced damage to both photosystem II and photosystem I
Source: Sci Rep. 2020 Apr 21;10:6770. doi: 10.1038/s41598-020-62717-1 (PMC7174426; doi:10.1038/s41598-020-62717-1)
Supplement: Supplementary file 1 — Supplementary Information. [file 41598_2020_62717_MOESM1_ESM.pdf]

Higher order photoprotection mutants reveal the importance of ΔpH-dependent photosynthesis-control in preventing light induced damage to both photosystem II and photosystem I

Roberto Barbato[1,*], Luca Tadini[2], Romina Cannata[1], Carlotta Peracchio[2], Nicolaj Jeran[2], Alessandro Alboresi[3], Tomas Morosinotto[3], Azfar Ali Bajwa[4], Virpi Paakkarinen[4], Marjaana Suorsa[4], Eva Mari Aro[4], Paolo Pesaresi[2]

[1]Department of Sciences and Innovation Technology, University of Eastern Piedmont, I-15121 Alessandria, Italy

[2] Department of Biosciences, University of Milan, I-20133, Milan, Italy

[3] Department of Biology, University of Padova, 35121 Padova, Italy

[4] Molecular Plant Biology, Department of Biochemistry, University of Turku, SF-20520 Turku, Finland

[*]correspondence: roberto.barbato@uniupo.it

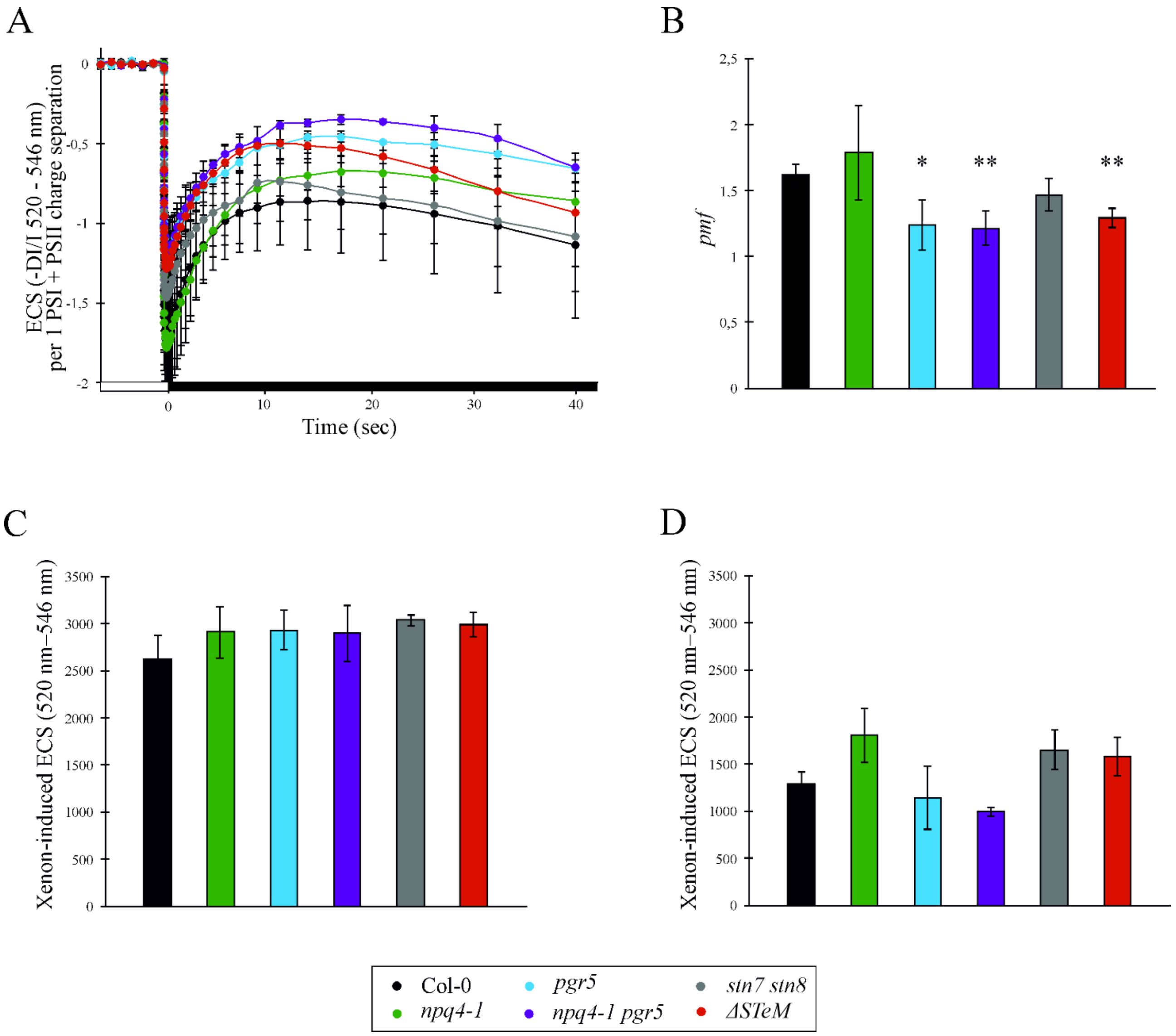

Supplementary Figure 1

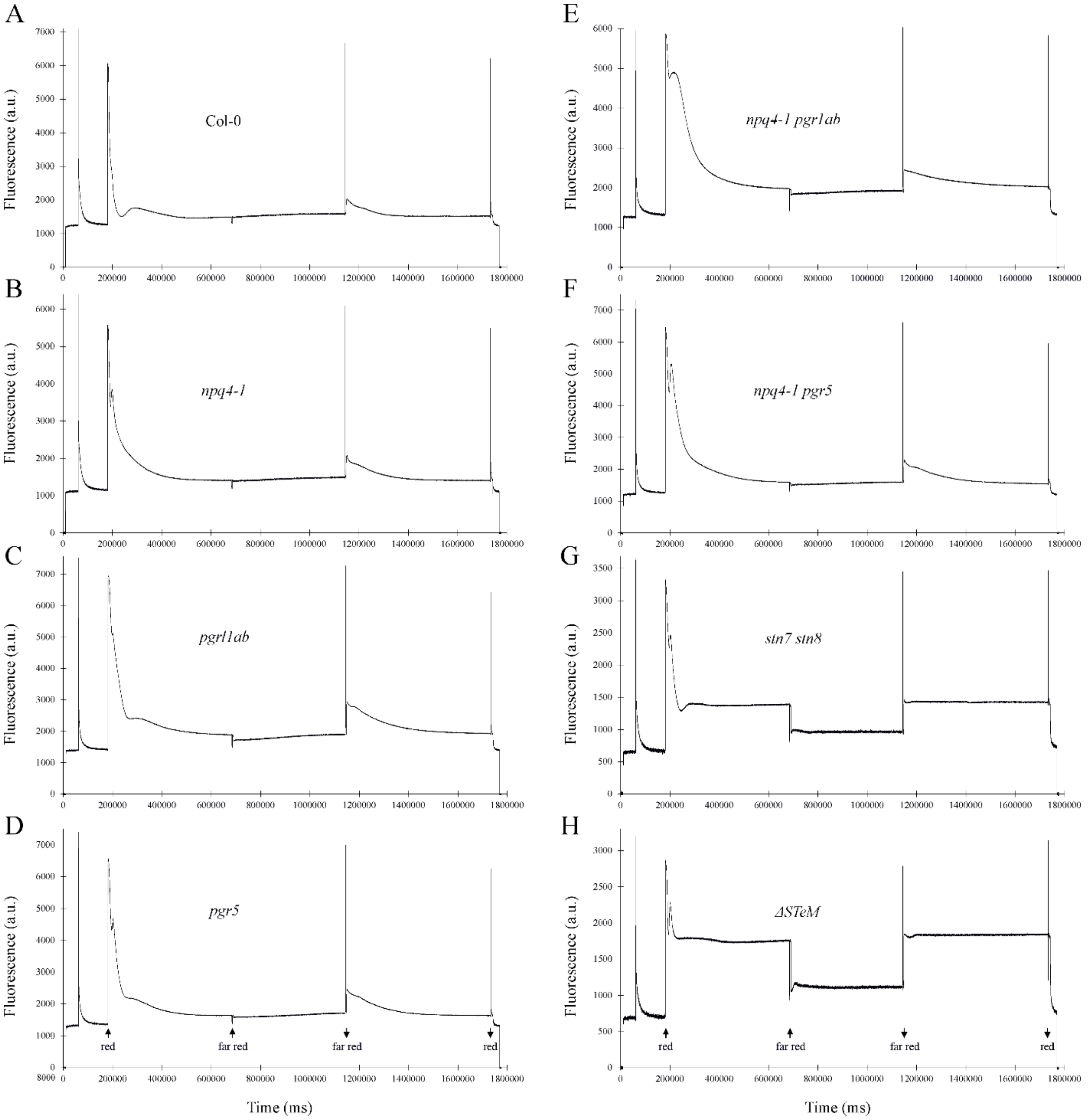

Supplementary Figure 2

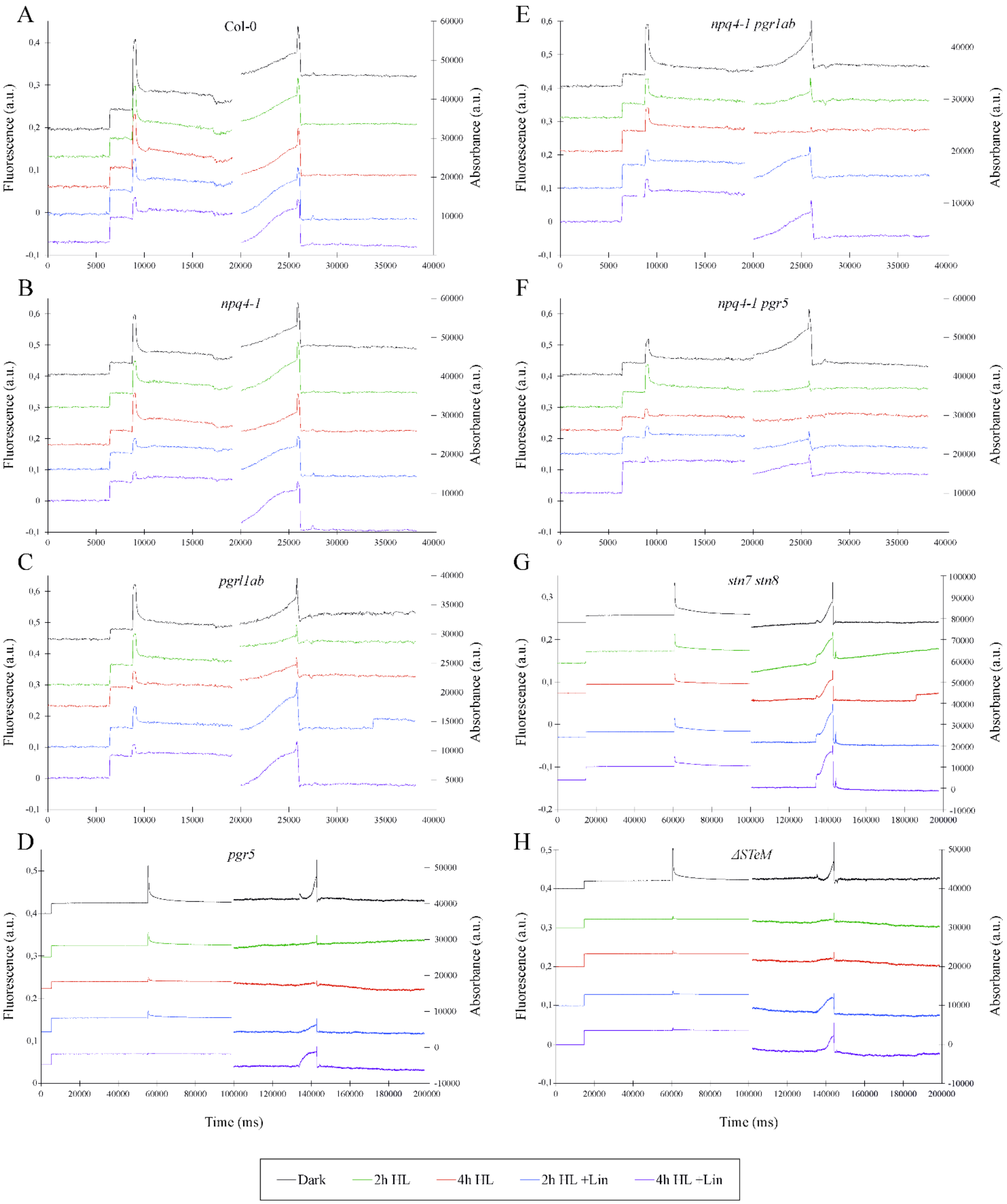

Supplementary Figure 3
